# The Effect of the Clinical-Pathological CPS+EG Staging System on Survival Outcomes in Patients with HER2-Positive Breast Cancer Receiving Neoadjuvant Treatment: A Retrospective Study

**DOI:** 10.3390/medicina61101813

**Published:** 2025-10-09

**Authors:** Seval Orman, Miray Aydoğan, Oğuzcan Kınıkoğlu, Sedat Yıldırım, Nisanur Sarıyar Busery, Hacer Şahika Yıldız, Ezgi Türkoğlu, Tuğba Kaya, Deniz Işık, Seval Ay Ersoy, Hatice Odabaş, Nedim Turan

**Affiliations:** Department of Medical Oncology, Health Science University, Kartal Dr Lutfi Kırdar City Hospital, Istanbul 34865, Türkiye; mirayaydogan1991@gmail.com (M.A.); ogokinikoglu@yahoo.com (O.K.); rezansedat@hotmail.com (S.Y.); sariyarnisanur@gmail.com (N.S.B.); h.sahikayildiz@gmail.com (H.Ş.Y.); ezgiturk_90@hotmail.com (E.T.); tugbakaya89@hotmail.com (T.K.); dnz.1984@yahoo.com (D.I.); drsevalay@gmail.com (S.A.E.); odabashatice@yahoo.com (H.O.); turan.nedim@hotmail.com (N.T.)

**Keywords:** HER2-positive breast cancer, CPS+EG score, neoadjuvant chemotherapy, survival, prognostic factors

## Abstract

*Background and Objectives*: To evaluate the prognostic value of the Clinical–Pathologic Stage–Estrogen receptor status and Grade (CPS+EG) staging system, which combines clinical staging, pathological staging, oestrogen receptor (ER) status, and tumour grade in predicting survival outcomes in patients with human epidermal growth factor receptor 2 (HER2)-positive breast cancer receiving neoadjuvant therapy (NACT). *Materials and Methods*: A retrospective review was performed on 245 female breast cancer patients who received anti-HER2 therapy alongside NACT at the Medical Oncology Department of Kartal Dr Lütfi Kırdar City Hospital, University of Health Sciences, from April 2012 to June 2024. The CPS+EG score was calculated using the MD Anderson Cancer Centre neoadjuvant treatment response calculator. Patients were categorised into two groups based on their CPS+EG score < 3 and ≥3. The primary outcomes assessed were disease-free survival (DFS) and overall survival (OS). Kaplan–Meier and log-rank tests were utilised for time-to-event analysis; Cox regression was used for multivariate analysis. A significance level of ≤0.05 was considered. *Results*: The median age of the patient cohort was 51 years (range: 27–82 years). Among these patients, 183 (74.6%) had a CPS+EG score less than 3, while 62 (25.3%) exhibited a score of 3 or higher. The median follow-up duration was 37.6 months. The pathological complete response (pCR) rate across the entire cohort was 51.8%. Specifically, the pCR rate was 56.3% in the group with CPS+EG scores below 3, and 38.7% in those with scores of 3 or higher (*p* = 0.017). Patients with CPS+EG scores less than 3 demonstrated superior overall survival (OS), which reached statistical significance in univariate analysis. Multivariate analysis identified the CPS+EG score as an independent prognostic factor for both overall survival and disease-free survival (DFS), with hazard ratios of 0.048 (95% CI: 0.004–0.577, *p* = 0.017) and 0.35 (95% CI: 0.14–0.86, *p* = 0.023), respectively. *Conclusions*: The CPS+EG score is an independent and practical prognostic marker, particularly for overall survival, in patients with HER2-positive breast cancer who have received neoadjuvant therapy. Patients with a CPS+EG score < 3 have higher pCR rates and survival rates. When used in conjunction with pCR, it can improve risk categorisation and contribute to the individualisation of adjuvant strategies in the post-neoadjuvant period. Due to its ease of calculation and lack of additional costs, this score can be instrumental in clinical practice for identifying high-risk patients. Our findings support the integration of the CPS+EG score into routine clinical decision-making processes, although prospective validation studies are necessary.

## 1. Introduction

Breast cancer is the most common malignant tumour in women and a leading cause of death. HER2-positive breast cancer, characterised by overexpression of the human epidermal growth factor receptor 2, tends to be more aggressive. This subtype accounts for about 20% of all breast cancer cases. The HER2 status is determined through immunohistochemical analysis, and the use of HER2-targeted monoclonal antibodies is a vital part of standard treatment regimens [1].

Neoadjuvant chemotherapy is a standard clinical approach in terms of tumour size reduction and an increase in breast-conserving surgery rates [2]. Although it is applied in local advanced breast cancer and early-stage breast cancer, it also allows for the assessment of the tumours’ in vivo treatment response. The response to neoadjuvant therapy provides important prognostic information and can guide adjuvant treatment in HER2-amplified and triple-negative breast cancers (TNBCs) with residual disease [3]. Pathological complete response, which is the absence of invasive residual tumour cells in the breast and axillary lymph nodes, is strongly associated with improved disease-free and overall survival, especially in the more aggressive subtypes of HER2-positive and triple-negative breast cancer [4]. This relationship is the most significant in triple-negative breast cancer. Adding anti-HER2 therapies to neoadjuvant chemotherapy in HER2-positive breast cancer approximately doubles the pathological complete response (pCR) rate. In particular, HER2-positive and triple-negative patients who achieve a pathological complete response after neoadjuvant treatment have better disease-free survival compared to those with residual disease [2].

Post-neoadjuvant treatment strategies are determined based on the response assessment conducted after neoadjuvant chemotherapy [4]. Studies conducted emphasise the significance of tumour biology beyond pathological complete response in prognostic evaluations. The CPS+EG staging system, developed for prognosis determination after neoadjuvant chemotherapy, enables a comprehensive review that considers not only the pathological stage but also the oestrogen status, nuclear grade, and clinical stage before treatment. It predicts five-year distant metastasis-free survival and disease-specific survival by scoring patients from 0 to 6 [5]. This score was initially developed and independently validated in the overall population of patients with breast cancer, regardless of tumour subtype [6].

Numerous studies have been conducted to assess prognosis in patients with residual disease after neoadjuvant chemotherapy, through pathological evaluations of disease burden and/or the biological features of the primary tumour [7,8].

One of the limitations associated with the development of the CPS+EG staging system is its prerequisite implementation before the routine administration of Trastuzumab in the management of HER2-positive breast cancer patients. The addition of Trastuzumab to neoadjuvant chemotherapy protocols results in significantly increased pathological complete response (pCR) rates compared to chemotherapy alone. Evidence indicates that achieving pCR through neoadjuvant chemotherapy combined with Trastuzumab in patients with HER2-positive breast cancer correlates with improved disease-free survival (DFS) and overall survival (OS) outcomes relative to patients who do not attain pCR [9,10,11,12].

The CPS+EG score offers prognostic information, especially in hormone receptor-positive, HER2-negative breast cancer, and aids in determining adjuvant treatment strategies. Its impact on various breast cancer subtypes remains uncertain [13,14,15].

The primary aim of this study was to assess the prognostic significance of the CPS+EG score on disease-free survival (DFS) and overall survival (OS) in patients with HER2-positive breast cancer who received neoadjuvant therapy. Patients were stratified into low-risk (<3) and high-risk (≥3) groups based on the CPS+EG score, which includes clinical stage, pathological stage, oestrogen receptor (ER) status, and tumour grade. The independent predictive value of this scoring system for survival outcomes was examined. The secondary aims involved evaluating the relationship between the CPS+EG score and pathological complete response (pCR), analysing survival differences in patients who did not achieve pCR according to their CPS+EG scores, and comparing the prognostic performance of the score with biological markers such as the ki-67 proliferation index. Additionally, the study aimed to explore the clinical usefulness of the CPS+EG system and its potential role in guiding adjuvant treatment strategies for high-risk patients.

## 2. Materials and Methods

### 2.1. Patient Population

A total of 245 female patients diagnosed with breast cancer, who were monitored between April 2012 and June 2024 at the Medical Oncology Clinic of Kartal Dr. Lütfi Kırdar City Hospital, University of Health Sciences, and who received neoadjuvant chemotherapy in combination with anti-HER2 therapy, were included in the study. The medical records of patients over the age of 18 with HER2-positive tumours were retrospectively reviewed. At the time of diagnosis, clinical staging was performed using ultrasound and mammography. The clinical staging of the patients was classified according to the TNM anatomic staging system for breast carcinoma, as per the 8th edition of the AJCC/UICC guidelines [16].

**Figure 1:**
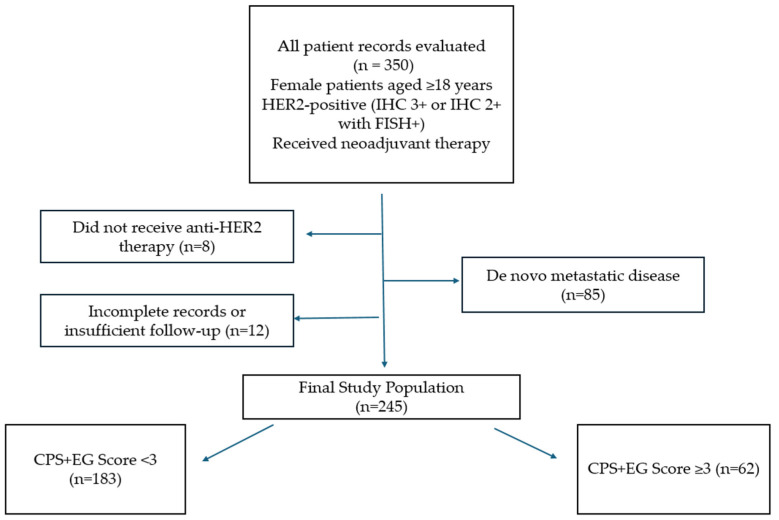
Flowchart of patient selection.

HER2 status was determined by immunohistochemical (IHC) staining. Tumours with a score of 3+ were considered HER2-positive. Tumours with a score of 2 for HER2 expression were subsequently analysed using fluorescence in situ hybridisation (FISH), and if HER2 amplification was present in the FISH test, they were considered HER2-positive. A HER2 FISH-positive result was defined as a HER2/CEP17 ratio of ≥2.0 or an average HER2 copy number of 6.0 signals per cell. Nuclear staining for oestrogen (ER) and progesterone (PR) receptors was considered positive if it was 1% or more, and ER and/or PR positivity was determined by IHC assessment by the guidelines of the American Society of Clinical Oncology and the College of American Pathologists (ASCO/CAP). The HER2 and ER status, as well as nuclear grade, were recorded based on the biopsy material obtained at diagnosis; the pathological stage was recorded based on the surgical material obtained after surgery (Figure 1).

The CPS+EG score, which integrates pre-treatment clinical staging, estrogen receptor status, tumour grade, and post-treatment pathological staging, was quantified utilising the MD Anderson Cancer Centre Neoadjuvant Treatment Response Calculator, with scores spanning from 0 to 6. In investigations evaluating the utility of the CPS+EG score in informing adjuvant treatment strategies, a score of less than 3 was classified as indicative of low risk [13,14].

### 2.2. Data Collection and Work Termination Points

The clinical and pathological features of the patients, including age, menopausal status, clinical stage of the tumour, levels of oestrogen and progesterone receptors, nuclear grade, HER2 status, CPS+EG scores, neoadjuvant chemotherapy regimens received, dates of surgery, post-operative pathological stages, adjuvant treatments, whether adjuvant radiotherapy was administered, and oncological outcomes, were recorded. The impact of the CPS+EG staging system on disease-free survival and overall survival, as a measure of prognosis, is the primary endpoint of the study. Disease-free survival (DFS) is defined as the period between the exact date of surgery and the first event. Overall survival (OS) is considered the time from the date of diagnostic core biopsy to death from any cause. In this study, pathological complete response (pCR) was defined by histopathological examination of the specimen after surgery performed following neoadjuvant therapy. Pathological complete response (pCR) was defined as the absence of invasive carcinoma in the breast and axillary lymph nodes (ypT0/is, ypN0). The presence of ductal carcinoma in situ (DCIS) in the breast was permitted. Lymph nodes containing only isolated tumour cells (ITCs, ≤0.2 mm, ≤200 cells) were classified as node-negative [ypN0(i+)].

### 2.3. Treatment Protocols and Surgery

The patients included in the study received the AC protocol: doxorubicin (60 mg/m^2^) and cyclophosphamide (600 mg/m^2^) every two or three weeks, followed by 12 weeks of weekly paclitaxel (80 mg/m^2^) or every three weeks for four cycles of docetaxel (75 mg/m^2^). Anti-HER2 treatments, including trastuzumab (8 mg/kg loading, 6 mg/kg maintenance) and/or pertuzumab (4 cycles; 840 mg loading, 420 mg maintenance), were started concurrently with the taxane chemotherapy regimen and administered every three weeks as neoadjuvant therapy. All patients underwent surgery after neoadjuvant treatment. Postoperative adjuvant trastuzumab was completed over a year, with a total of 17 cycles administered. Trastuzumab emtansine (T-DM1) received approval in Turkey in 2021, and some patients with residual disease postoperatively were treated with up to 14 cycles of T-DM1.

### 2.4. Scoring System

The CPS+EG score was determined using the MD Anderson Neoadjuvant Treatment Response Calculator, which combines four clinical and pathological variables: clinical stage before neoadjuvant therapy, pathological stage after surgery, oestrogen receptor (ER) status, and nuclear grade. Points are allocated as follows (Table 1):

The total score ranges from 0 to 6. Based on the original MD Anderson model, patients were stratified into two prognostic categories [17]:

- Low-risk group: CPS+EG < 3
- High-risk group: CPS+EG ≥ 3

### 2.5. Statistical Analysis

SPSS (version 25.0, IBM Corp. Armonk, NY, USA, 2013) was utilised for statistical analysis. Descriptive statistics were performed, and categorical data were reported as frequencies (percentages). For numerical variables between two independent groups, Student’s *t*-test was applied if the data were normally distributed; otherwise, the Mann–Whitney U test was used. Univariate and multivariate analyses were conducted using a logistic regression model to evaluate prognostic factors affecting pathological complete response. The effect of prognostic factors on pathological complete response was assessed using univariate log-rank tests. The hazard ratio (HR) was calculated with a 95% confidence interval (CI). Multivariate analysis was performed using Cox regression modelling to evaluate the impact of prognostic factors on pathological complete response. Event-time analyses were conducted for DFS and OS, and Kaplan–Meier estimates were plotted. The log-rank test was utilised to compare DFS between groups. The significance level was set at ≤0.05. The median follow-up time was estimated using the reverse Kaplan–Meier method.

Variables that were statistically significant in univariate analyses (*p* < 0.05) and those considered clinically relevant (clinical T stage, nodal status, ER status, pCR) were included in the multivariate Cox regression model using forced entry. The proportional hazards assumption was evaluated with Schoenfeld residuals, and no violations were identified.

## 3. Results

A total of 245 patients were included in this study. The median age was 51 years (range: 27–82 years). Of these participants, 109 (44.5%) were in the premenopausal or perimenopausal phase, while 136 (55.5%) were postmenopausal. At diagnosis, disease staging revealed that 170 patients (69.7%) were classified as stage II, 57 (23.4%) as stage III, and 17 (7%) as stage I. Before neoadjuvant therapy, 42 patients (17.1%) exhibited a HER2 score of 2 (FISH-positive), whereas 203 patients (82.9%) demonstrated a HER2 score of 3. Regarding nodal status, 42 patients (17.1%) were N0, 156 (63.7%) N1, 39 (15.9%) N2, and 8 (3.3%) N3. Patients were stratified according to the CPS+EG score into two groups: those with scores below three and those with scores of 3 or higher. The cohort with CPS+EG scores < 3 Comprised 183 patients (74.6%), while 62 patients (25.3%) had scores ≥ 3. Among patients with CPS+EG scores < 3, 68.3% exhibited nuclear grade 2, whereas only 16.9% of those with scores ≥ 3 had nuclear grade 2. Conversely, 31.7% of patients with scores < 3 had nuclear grade 3, compared to 83.1% in the group with scores ≥ 3. This difference was statistically significant (*p* < 0.01) (Table 2).

The median follow-up duration for the patients included in the study was determined to be 37.6 months using the reverse Kaplan–Meier method (95% CI 33.5–41.8). The pathological complete response rate across the entire cohort was 51.8% (95% CI, 45.5–58.1%). Among patients with a CPS+EG score < 3, 56.3% (95% CI, 49.2–63.4%) achieved a pathological complete response, whereas 38.7% (95% CI, 25.4–52.0%) of those with a CPS+EG score ≥ 3 did so; this difference was statistically significant (*p* = 0.017).

**Figure 2:**
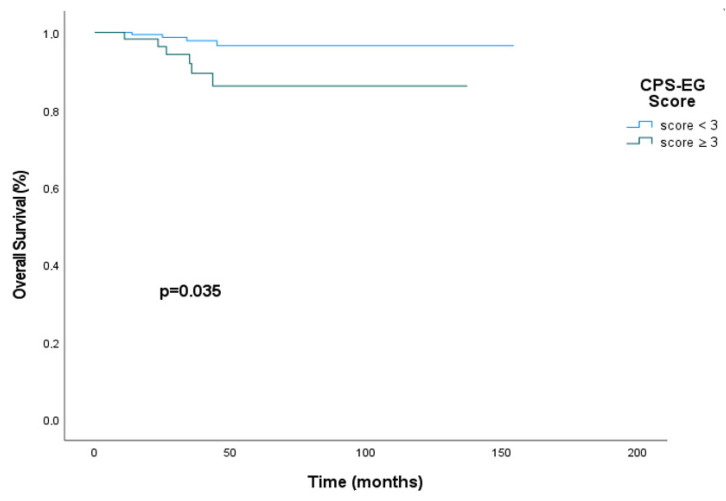
Overall survival curves stratified by the CPS+EG score in the entire HER2-positive cohort receiving neoadjuvant therapy.

The median overall survival could not be reached for patients with a CPS+EG score < 3. However, at the 50th month, 96.7% (95% CI, 92.0–100.0%) of patients with a CPS+EG score < 3 were alive, compared to 88.1% (95% CI, 78.1–98.1%) of patients with a CPS+EG score ≥ 3. In the univariate analysis, patients with a CPS+EG score below 3 had a statistically significantly more prolonged overall survival, but median overall survival could not be reached in either group (HR: 0.27; 95% CI, 0.072–1.00; *p* = 0.035) (Figure 2).

**Figure 3:**
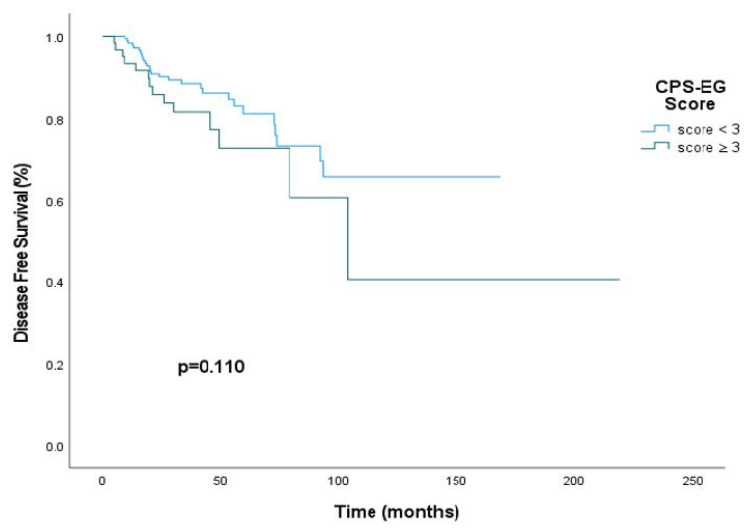
Disease-free survival (DFS) curves stratified by CPS+EG scores within the entire cohort of HER2-positive patients receiving neoadjuvant therapy.

The disease-free survival (DFS) rate at 50 months was 86.4% (95% CI, 77.4–95.4%) in patients with a CPS+EG score less than 3, compared to 61.2% (95% CI, 39.1–83.3%) in patients with a CPS+EG score of 3 or higher. The median DFS could not be determined in the cohort with CPS+EG scores below 3. For patients with a CPS+EG score of 3 or greater, the median DFS was 103.9 months (interquartile range: 59.5–148.3 months). However, this difference did not reach statistical significance (HR: 0.60; 95% CI, 0.31–1.13; *p* = 0.110) (Figure 3).

**Figure 4:**
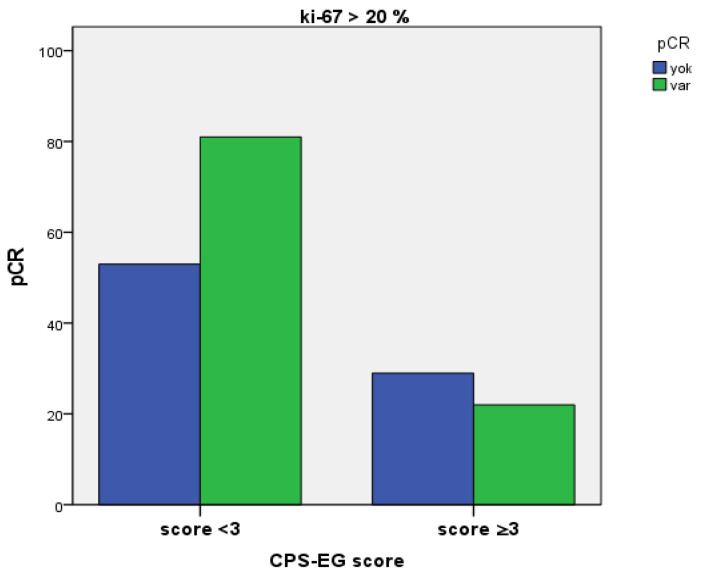
Rates of Pathological Complete Response Stratified by CPS+EG score in patients exhibiting a Ki-67 proliferation index over 20%.

While 60% of patients exhibiting a pre-treatment Ki-67 proliferation index greater than 20% and a CPS+EG score less than 3 achieved pathological complete response (95% CI, 52.4–67.6%), a statistically significant pCR rate of 43.1% (95% CI, 29.6–56.6%) was observed in patients with a CPS+EG score of 3 or higher (*p* = 0.046) (Figure 4).

**Figure 5:**
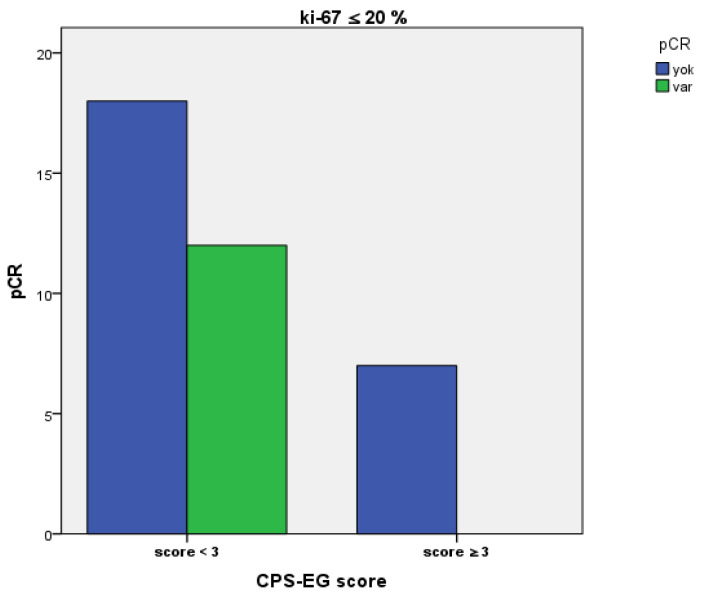
Rates of pathological complete response stratified by CPS+EG score in patients with a Ki-67 proliferation index of ≤20%.

While 40% of patients with a pre-treatment Ki-67 proliferation index of ≤20% and CPS+EG score below 3 achieved a pathological complete response, no pCR was observed in those with a CPS+EG score of 3 or higher (*p* = 0.072) (Figure 5). We included the parameters that were significant in univariate analyses and could clinically contribute to DFS and OS in the multivariate analysis. It was found that the CPS+EG score had a statistically significant impact on overall survival in both univariate and multivariate analyses (respectively, *p* = 0.022, *p* = 0.017). In multivariate analysis, CPS+EG ≥ 3 served as the reference group, whereas CPS+EG < 3 demonstrated significantly better OS (HR 0.048; 95% CI: 0.004–0.577). Although the HR appears numerically extreme, it reflects the substantial survival benefit in the low-risk group within our cohort, as also indicated by the wide confidence interval. For DFS, CPS+EG was not significant in univariate analysis but became significant after multivariate adjustment.

When assessing the factors influencing overall survival, the CPS+EG score was identified as being statistically significant in both univariate and multivariate analyses (*p* = 0.017, HR = 0.048, 95% CI: 0.004–0.57) (Table 3). Additionally, it was observed that the clinical T stage exhibited a nearly significant effect on overall survival. (*p* = 0.051 HR:0.19 CI (0.036–1.005).

In univariate analysis, the CPS+EG score had no significant impact on DFS, but in multivariate analysis, a substantial effect of the CPS+EG score on disease-free survival was observed. (HR: 0.35, 95% CI, 0.14–0.86; *p* = 0.023) (Table 4).

**Figure 6:**
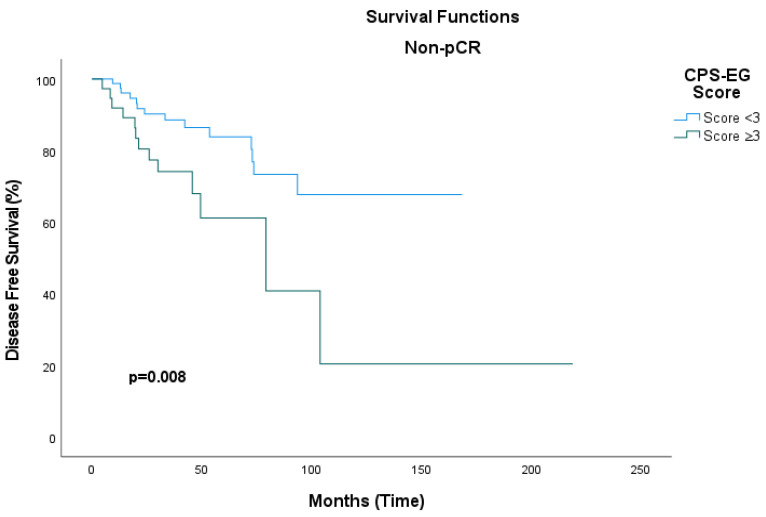
Disease-free survival curve stratified by CPS+EG score in patients without pathological complete response (non-pCR).

Among patients who achieved a pathological complete response, the median disease-free survival (DFS) was not reached in either group, and no statistically significant difference was observed (hazard ratio [HR]: 0.31; 95% confidence interval [CI], 0.04–2.35; *p* = 0.26). In patients with non-pCR who had CPS+EG scores below 3, the median DFS could not be determined; conversely, in the subgroup with CPS+EG scores of 3 or greater, the median DFS was 79.2 months (95% CI, 27.7–130.9). This difference was statistically significant (HR: 2.7; 95% CI, 1.26–5.85; *p* = 0.011) (Figure 6).

## 4. Discussion

This retrospective study aims to assess the prognostic value and survival implications of the CPS+EG score in women diagnosed with HER2-positive breast cancer, regardless of hormone receptor status, who are undergoing neoadjuvant chemotherapy (NACT). Developing accessible and reliable tools to evaluate long-term prognosis in this patient group, who receive NACT, is of vital importance [7].

The CPS+EG score constitutes a staging system developed to enhance the precision of long-term survival prognostications beyond the conventional AJCC tumour staging framework. It incorporates histopathological tumour characteristics, including hormone receptor status and nuclear grade [13,15,17].

The findings of this study indicate that patients with a CPS+EG score below 3 exhibit higher rates of pathological complete response (pCR) as well as improved overall survival (OS). It is well established that achieving pCR is associated with favourable long-term survival outcomes; however, our results suggest that the CPS+EG score serves as an independent prognostic marker for survival, providing predictive value beyond pCR status. Furthermore, the existing literature corroborates the significant predictive capacity of the CPS+EG score for both overall survival (OS) and distant metastasis-free survival (DMFS) across different breast cancer subtypes, including HER2-positive and triple-negative breast cancer (TNBC) [18]. Our study supports these findings. In patients with a CPS+EG score ≥ 3, the pCR rate was lower, nuclear grade was higher, nodal involvement was more extensive, and overall survival was significantly worse in these patients (*p* = 0.035). In multivariate analysis, the CPS+EG score was identified as an independent prognostic factor for OS and DFS (HR: 0.048; *p* = 0.017; HR: 0.35; *p* = 0.023).

Whether a pathological complete response is achieved or not, numerous research groups utilise various assessment tools to evaluate prognosis following neoadjuvant chemotherapy [8]. Among these diagnostic tools, the Residual Cancer Burden (RCB) classification demonstrates superior accuracy compared to the pAJCC staging system in precisely predicting the risk of postoperative recurrence [19]. One limitation of our study is the lack of RCB assessment for the included cases. The small sample size further limits the generalisability of the findings.

The CPS+EG score development team has incorporated not only the pathological AJCC stage of the operative specimen but also the clinical AJCC tumour stage, estrogen receptor (ER) expression, and tumour nuclear grade. This comprehensive approach facilitates prognostication in patients undergoing neoadjuvant chemotherapy (NACT) and enables the identification of candidates suitable for additional adjuvant therapies. Subsequent investigations have further evaluated its efficacy across specific breast cancer subtypes. The score has demonstrated reliability in prognostic assessment, particularly in HER2-positive breast tumours [20].

The CPS+EG score has been employed in multiple studies to stratify patients according to risk categories. In the OlympiA trial, the efficacy of adjuvant olaparib was examined in patients with early-stage HER2-negative tumours and germline BRCA mutations, with high-risk patients defined as those exhibiting a CPS+EG score greater than 3 [21]. In the PENELOPE-B trial, the efficacy of adjuvant palbociclib was assessed in patients with hormone receptor-positive, HER2-negative breast cancer who exhibited residual disease following neoadjuvant chemotherapy. High-risk classification was determined based on a CPS+EG score of 3 or higher, or the presence of ypN1 nodal status [22].

Adjuvant administration of sacituzumab govitecan (SG), an antibody–drug conjugate targeting Trop-2, was also utilised in the ongoing phase 3 randomised SASCIA trial. This intervention aimed to identify patients at high risk of relapse after surgical intervention, based on the CPS+EG score [23].

Pathological complete response is a parameter that has a strong relationship with DFS and OS, particularly in patients with hormone-negative breast cancer undergoing neoadjuvant chemotherapy [6,24]. Although our primary aim was to assess the prognostic value of the CPS+EG score in HER2-positive patients regardless of hormone receptor status, we also evaluated pCR since our patients underwent neoadjuvant chemotherapy. In our overall cohort, the rate of patients achieving a pathological complete response was 51.8%, which aligns with the literature. Typically, this rate in HER2-positive patients ranges approximately between 45% and 60% [6,25,26].

In univariate analyses, the CPS+EG score, a prognostic indicator for overall survival, was also found to be predictive of 50-month overall survival in multivariate models. While further comprehensive studies are required in this domain, our findings suggest that the CPS+EG score serves as an effective prognostic biomarker comparable to pathologic complete response (pCR).

The Neo-Bioscore, an evolved iteration of the CPS+EG score, aims to augment prognostic accuracy by integrating HER2 status and nuclear grade. This scoring system encompasses eight parameters, including pathological stage, clinical stage, estrogen receptor (ER) status, nuclear grade, and HER2 status. Notably, while Neo-Bioscore demonstrates superior discriminatory capacity in HER2-negative patient subsets, its additional prognostic value in HER2-positive patients appears limited [20]. In our study, the evaluation was limited to patients with HER2-positive breast cancer. Within this subgroup, the CPS+EG score was found to be an independent and significant prognostic indicator for overall survival.

The post-neoadjuvant (post-surgery) T-DM1 era further grounds these scores in clinical decision-making. The KATHERINE trial demonstrated that, among HER2-positive patients with residual invasive disease after NACT, adjuvant T-DM1 reduced the risk of invasive events or death by approximately 50% and maintained the invasive disease-free survival and overall survival benefits on long-term follow-up compared to trastuzumab. These results highlight the clinical importance of better defining a “high-risk” phenotype in patients without PCR; they also indicate that CPS+EG/Neo-Bioscore can improve post-NACT risk classification and assist in identifying candidates for intensification strategies such as T-DM1 [27]. In practice, CPS+EG/Neo-Bioscore, due to its ease of calculation and broad familiarity, can be positioned alongside quantitative pathologic measures, such as RCB, as part of a multilayered risk-stratification approach. Such a framework may rationalise post-neoadjuvant intensification (e.g., T-DM1) for non-pCR patients while helping to avoid overtreatment in those who achieve pCR [19].

In recent years, it has been reported that HER2 expression provides crucial prognostic information not only in invasive tumours but also in cases of ductal carcinoma in situ (DCIS). The literature shows that HER2 positivity in DCIS is associated with high histological grade, increased proliferative activity, and multifocality. Additionally, it has been reported that patients with HER2-positive DCIS have a higher risk of local recurrence, particularly an increased likelihood of invasive recurrence. Therefore, assessing HER2 expression is clinically significant not only in invasive cancers but also in DCIS, for predicting biological behaviour and guiding adjuvant treatment strategies. Indeed, some studies have suggested that using anti-HER2 targeted agents in HER2-positive DCIS cases may reduce the risk of recurrence. These findings regarding HER2 biology in DCIS may inform future personalised prognostic and therapeutic approaches [28,29,30]. In line with this concept, our results show that within invasive HER2-positive tumours, patients with lower CPS+EG scores (<3) achieved higher pathological complete response rates and better survival. Overall, both DCIS data and our findings in invasive disease emphasise the consistent prognostic significance of HER2 expression, highlighting the importance of incorporating HER2 status into risk stratification and post-neoadjuvant treatment decisions.

Additionally, in a separate investigation involving patients with metastatic breast cancer treated with neoadjuvant epirubicin, gemcitabine, and docetaxel irrespective of molecular subtype, the CPS+EG score emerged as the most significant prognostic factor for disease-free survival (DFS) in both univariate (*p* = 0.0006) and multivariate analyses. The hazard ratios (HRs) associated with the CPS+EG score ranged from 0.18 to 0.45 across different score thresholds (For CPS+EG scores ranging from 0 to 4), with a significance level of *p* < 0.002 [31]. In our study, the CPS+EG score was also identified as an important prognostic factor in the multivariate analysis for DFS (HR: 0.35, *p* = 0.023).

An additional point worth noting is the apparent discrepancy between univariate and multivariate DFS results. While CPS+EG did not reach statistical significance in the univariate model, it became significant in the multivariate analysis. This pattern likely indicates a suppression effect caused by correlations between CPS+EG components and baseline stage, nodal burden, and treatment variables. Once these overlapping predictors were considered, the independent prognostic impact of CPS+EG on DFS became more apparent. Such divergence between univariate and multivariate findings has been described in previous prognostic modelling studies and emphasises the importance of considering related clinical variables.

In our study, the relationship between the CPS+EG score and the pre-treatment Ki-67 proliferation index was assessed. Patients with a CPS+EG < 3 exhibited a significantly higher pCR rate (*p* = 0.046), and this finding suggests that the CPS+EG score may indicate not only the anatomical characteristics but also the biological behaviour of the tumour. Although it is well established in the literature that a high Ki-67 index correlates with poor prognostic indicators [32], there is no standard cut-off value in clinical practice. Generally, a range of 10–20% is used [33]. In our analysis, evaluations performed with a 20% cut-off value did not identify the Ki-67 index as an independent prognostic factor (*p* = 0.783 and *p* = 0.255). In subgroup analyses, pCR was 40% in patients with CPS+EG < 3 and Ki-67 ≤ 20%, whereas no pCR was achieved in those with CPS+EG ≥3. However, the small number of patients with Ki-67 > 20% may have contributed to this difference [34,35].

Recent research has expanded beyond traditional proliferation markers to include novel molecules that reflect the tumour microenvironment and biological behaviour. Within this framework, chemerin expression has been identified as being associated with enhanced tumour aggressiveness in breast cancer cells. A recent investigation demonstrated that elevated chemerin expression correlates with increased proliferative capacity, higher histological grade, and greater invasive potential. These findings underscore the significance of the inflammatory microenvironment and adipokine signalling pathways in tumour progression [36]. Although our study does not directly assess chemerin levels, our findings suggest that CPS+EG already reflects some aspects of tumour biology beyond anatomical staging, as patients with higher scores (≥3) had worse survival outcomes and lower pCR rates. Integrating biomarkers such as chemerin into prognostic scoring systems like CPS+EG may enhance the accuracy of risk stratification models, thereby facilitating the identification of high-risk HER2-positive patients who could potentially benefit from more aggressive post-neoadjuvant therapeutic strategies.

The CPS+EG score represents a simple, cost-effective, and easily accessible prognostic tool that has significant value in assessing the response to neoadjuvant chemotherapy. It applies not only to patients who do not achieve pathological complete response (pCR) but also broadly across the patient population for survival forecasting. Notably, the CPS+EG score may serve as a helpful guide in directing high-risk patients towards adjuvant therapy. Furthermore, the CPS+EG staging system can be integrated with molecular biomarkers and advanced imaging techniques to improve risk stratification and support the development of personalised treatment strategies in breast cancer management. Future research should focus on exploring these integrative approaches to optimise patient outcomes.

### 4.1. Limitations

This study has several limitations. First, we did not calculate the Residual Cancer Burden (RCB) index; as a result, we could not directly compare CPS+EG with RCB or the post-neoadjuvant AJCC (pAJCC) pathological staging system. This prevented a direct assessment of whether a combined clinicopathological score (CPS+EG) or a quantitative residual disease metric (RCB) offers better discrimination and calibration in HER2-positive disease after NACT. Second, by design, our focus was on CPS+EG, and we did not recalculate or compare Neo-Bioscore within our cohort, which limits conclusions about the added value of including HER2 status in our dataset. Third, this was a single-centre, retrospective analysis with a modest sample size, which reduces generalisability and increases the risk of unmeasured confounding. Fourth, variation in treatment across the study period may have affected outcomes. All patients received anti-HER2 therapy with taxane-based NACT; however, Pertuzumab was approved in Türkiye in October 2019 for neoadjuvant treatment of HER2-positive breast cancer, and Trastuzumab emtansine (T-DM1) was approved in Türkiye in November 2021, while adjuvant pertuzumab is not yet approved. Consequently, patients treated in the later years of our cohort may have benefited from dual HER2 blockade or adjuvant T-DM1 in the post-neoadjuvant setting, introducing a potential “era effect.” However, survival models were not adjusted for treatment era or regimen type, and we also did not perform separate sensitivity analyses restricted to patients from the modern era. In our study, OS was measured from the time of the diagnostic biopsy, and DFS was calculated from the date of surgery. We recognise that this methodological difference may introduce bias in comparisons. Furthermore, as the median OS was not reached in either risk group, the statistical significance observed should be interpreted with caution. Fifth, the median follow-up of approximately 38 months may be insufficient to characterise late recurrences in hormone-receptor-positive disease entirely, and we did not perform formal model-performance analyses (e.g., time-dependent AUC/C-index, calibration plots, or decision-curve analysis), which would help benchmark CPS+EG against alternative prognostic frameworks. Additionally, for DFS, we observed a suppression pattern. CPS+EG was not significant in univariable analysis but became significant after multivariable adjustment, possibly because correlated covariates (e.g., clinical stage and anti-HER2 regimen) weakened the unadjusted association. The effect direction (HR < 1) was consistent across models. While such divergence is expected with intercorrelated predictors or uneven event distribution. This suppression effect likely arises from correlations among covariates such as clinical stage, nodal status, and treatment regimen, rather than overfitting. Moreover, we did not perform formal assessments of non-linearity (e.g., restricted cubic splines) or sensitivity analyses across alternative CPS+EG cut-points. While prior validation studies consistently support <3 versus ≥3 as a meaningful threshold, the absence of such exploratory analyses may limit the generalisability of our findings. Finally, no formal adjustment for multiple testing was conducted despite several subgroup and survival analyses; this is acknowledged as a limitation of our study.

### 4.2. Future Perspectives

Future research should prospectively incorporate CPS+EG, Neo-Bioscore, pAJCC, and RCB within the same HER2-positive NACT groups, enabling direct comparisons of discrimination, calibration, and clinical utility (including time-dependent AUC/C-index, net reclassification improvement, and decision-curve analysis). A multicentre, prospective study with central pathology review and a predefined collection of RCB components (residual primary size, cellularity, and nodal burden) will strengthen robustness and external validity. Considering the current post-T-DM1 context, analyses should be stratified by pCR versus non-pCR and by receipt of adjuvant T-DM1 to determine whether composite scores offer better guidance for treatment intensification or de-escalation beyond pCR alone. Furthermore, incorporating biological and microenvironmental markers (e.g., HER2-low/zero conversion, Ki-67, TILs, PIK3CA/TP53 status) alongside emerging circulating tumour DNA (ctDNA) minimal residual disease tests may support the development of multi-modal prognostic models that outperform single-parameter tools. Future studies with larger cohorts and standardised Ki-67 assessment should also model Ki-67 as a continuous variable and explore potential interactions with CPS+EG to refine risk stratification. Imaging-based response metrics (e.g., MRI tumour shrinkage patterns) could also enhance pathological assessments. In summary, integrating molecular biomarkers such as PIK3CA mutations, HER2 heterogeneity, ctDNA, and TILs into CPS+EG may enable the development of clinico-molecular models that refine risk stratification and more precisely guide escalation or de-escalation strategies in the modern post-neoadjuvant setting. Re-assessment of receptor status on residual disease, alongside the inclusion of circulating tumour DNA (ctDNA) and tumour-infiltrating lymphocytes (TILs), could further improve prognostic accuracy. Developing and validating such a composite model could offer a more precise tool for guiding escalation or de-escalation of therapeutic strategies in the post-neoadjuvant setting.

This pattern relates to confounding or suppression: baseline disease burden and treatment factors that correlate with components of CPS+EG can weaken its unadjusted association but reveal its independent effect when included in the multivariable model. In our dataset, covariates such as clinical stage and the type of neoadjuvant anti-HER2 regimen varied across CPS+EG strata. The neoadjuvant regimen showed a signal in univariable screening; therefore, adjustment for correlated predictors reduced interference and clarified the link between CPS+EG and DFS. Notably, the effect direction (HR < 1) remained consistent across models, supporting a suppression rather than a reversal effect. From a methodological perspective, such divergence between univariable and multivariable results is expected when predictors are correlated or when outcome events are unevenly distributed across risk groups. While our analysis followed a predefined adjustment strategy, we recognise that additional sensitivity analyses, such as checks of proportional hazards, assessment of collinearity (VIF), and parsimony relative to events-per-variable, would further improve interpretability and will be prioritised in future work.

## 5. Conclusions

This study shows that the CPS+EG score is a strong and independent marker for prognosis in patients with HER2-positive breast cancer treated with neoadjuvant chemotherapy. Patients with a CPS+EG score < 3 not only achieved higher rates of pathological complete response but also experienced better overall survival outcomes. Significantly, the CPS+EG score provides prognostic information beyond the pathological complete response and reflects both anatomical and biological features of the tumour. In the post–T-DM1 era, the CPS+EG score can help identify high-risk patients who would benefit from adjuvant intensification strategies, while supporting treatment de-escalation in low-risk groups. Although more quantitative indices such as RCB or Neo-Bioscore exist, the simplicity and cost-effectiveness of CPS+EG support its broad applicability in real-world clinical practice.

Due to its simplicity, cost-effectiveness, and ease of integration into routine clinical practice, the CPS+EG score could be a valuable tool for identifying high-risk patients who might benefit from more intensive treatments after neoadjuvant therapy. Larger-scale prospective studies are necessary to validate our findings and investigate how the CPS+EG score can be combined with molecular biomarkers and advanced imaging techniques. This approach may enhance risk stratification and improve patient outcomes in personalised breast cancer management.

The future integration of molecular biomarkers (e.g., Ki-67, ctDNA, TILs, and chemerin expression) and imaging-based response metrics could further enhance the discriminatory power of CPS+EG and promote the development of multi-parametric prognostic models.

## Figures and Tables

**Table 1:** Components and Scoring Criteria of the CPS+EG System.

Variable	Category	Points
Clinical stage (cStage)	cI	0
	cII	1
	cIII	2
Pathological stage (pStage)	p0–I	0
	pII	1
	pIII	2
Estrogen receptor (ER) status	Positive	0
	Negative	1
Nuclear grade	Grade 1–2	0
	Grade 3	1

**Table 2:** Demographic and Clinical Features of the Study Participants.

Variable	Total (*n* = 245)	CPS+EG Score < 3 (183)	CPS+EG Score ≥ 3 (62)	*p*-Value
Median age (years)				0.86
≤50	117 (47.8%)	88 (48.1%)	29 (46.8%)	
>50	128 (52.2%)	95 (51.9%)	33 (53.2%)	
Menopausal Status				0.68
Pre/perimenopause	109 (44.5%)	80 (43.7%)	29 (46.8%)	
Postmenopause	136 (55.5%)	103 (56.3%)	33 (53.2)	
ER status				<0.001
Positive	138 (43.7%)	124 (67.8%)	14 (22.6%)	
Negative	107 (56.3%)	59 (32.2%)	48 (77.4%)	
PR status				<0.001
Positive	113 (46.1%)	98 (53.6%)	15 (24.2%)	
Negative	132 (53.9%)	85 (46.4%)	47 (75.8%)	
Pretreatment Ki-67 Status				0.312
≤20	37 (16.7%)	30 (18.3%)	7 (12.1%)	
>20	185 (83.3%)	134 (81.7%)	51 (87.9%)	
Clinical Stage				<0.01
Stage 1	17 (7%)	17 (9.3%)	0 (0%)	
Stage 2	170 (69.7%)	133 (73.1%)	37 (59.7%)	
Stage 3	57 (23.4%)	32 (17.6%)	25 (40.3)	
pCR				0.017
Yes	127 (51.8%)	103 (56.3%)	24 (38.7%)	
No	118 (48.2%)	80 (43.7%)	38 (61.3%)	
HER2 Status				0.80
Score 2 FISH-positive	42 (17.1%)	32 (17.5%)	10 (16.1%)	
3+	203 (82.9%)	151 (82.5%)	52 (83.9%)	
Clinical N				<0.01
0	42 (17.1%)	41 (22.4%)	1 (1.6%)	
1	156 (63.7%)	116 (63.4%)	40 (64.5%)	
2	39 (15.9%)	24 (13.1%)	15 (24.2%)	
3	8 (3.3%)	2 (1.1%)	6 (9.7%)	
Grade				<0.01
2	124 (54.9%)	114 (68.3%)	10 (16.9%)	
3	102 (45.1%)	53 (31.7%)	49 (83.1%)	
NACT Type				0.65
AC+HP+Taxan	150 (61.2%)	110 (60.1%)	40 (64.5%)	
AC+H+Taxan	95 (38.8%)	73 (39.9%)	22 (35.5%)	

**Table 3:** Overall Survival Univariate and Multivariate Analysis.

	Univariate Analysis		Multivariate Analysis	
	HR, 95% CI	*p* Value	HR, 95% CI	*p* Value
Median age (years) ≤50 >50	1.901 (0.475–7.605)	0.364		
Menopausal status (%) Pre/Perimenopause Postmenopause	1.120 (0.300–4.174	0.866		
pCR Yes No	0.333 (0.069–1.607)	0.171	0.558 (0.102–3.056)	0.501
ER positivity	0.338 (0.084–1.353)	0.125		
PR positivity	0.335 (0.070–1.612)	0.172		
Pretreatment ki-67 ≤20% >20%	0.445 (0.111–1.782)	0.253	0.349 (0.057–2.136)	0.255
Grade 2 3	2.150 (0.514–9.001)	0.295	0.566 (0.084–3.830)	0.559
cT stage T1 T2 T3 T4	0.579 (0.192–1.743)	0.331	0.191 (0.036–1.005)	0.051
cN stage N1 N2 N3	0.887 (0.463–1.699)	0.717	1.279 (0.444–3.686)	0.649
Clinical stage Stage I Stage II Stage III	0.961 (0.560–1.650)	0.886		
CPS+EG score <3 ≥3	4.376 (1.234–15.517)	0.022	0.048 (0.004–0.577)	0.017
NACT type Dual Anti-HER2 vs. mono Anti-HER2	0.650 (0.167–2.528)	0.534		

**Table 4:** Disease-Free Survival Univariate and Multivariate Analysis.

	Univariate		Multivariate	
	HR (95% CI)	*p*-Value	HR (95% CI)	*p*-Value
CPS+EG Score	0.60 (0.31–1.13)	0.114	0.35 (0.14–0.86)	0.023
pCR	0.66 (0.35–1.25)	0.198	0.30 (0.03–2.67)	0.281
ER positivity	0.642 (0.350–1.177)	0.152		
PR positivity	0.576 (0.306–1.087)	0.089		
Pretreatment ki-67 ≤20% >20%	1.50 (0.45–4.95)	0.508	1.14 (0.46–2.80)	0.783
Grade	1.16 (0.71–1.92)	0.554	1.33 (0.70–2.51)	0.382
cT stage	1.16 (0.78–1.74)	0.464	1.49 (0.81–2.73)	0.195
cN stage	0.96 (0.73–1.25)	0.753	1.17 (0.77–1.80)	0.884

## Data Availability

The data shown in this study can be obtained by request from the corresponding author.

## Abbreviations

The following abbreviations are used in this manuscript:

NACT	Neoadjuvant Chemotherapy
HER2	Human epidermal growth factor receptor 2
CPS+EG	Clinical and pathologic stage, estrogen receptor status, and histologic grade
FISH	Fluorescence in situ hybridisation
DCIS	Ductal carcinoma in situ
pCR	Pathological complete response
ITC	Isolated tumour cells
ER	Estrogen receptor
ctDNA	circulating tumour DNA
TILs	tumour-infiltrating lymphocytes
IHC	Immunohistochemistry
HP	Trastuzumab + Pertuzumab
ASCO	American Society of Clinical Oncology
CAP	College of American Pathologists
AC	Doxorubicin and Cyclophosphamide
OS	Overall survival
DFS	Disease-free survival
HR	Hazard ratio
CI	Confidence interval
RCB	Residual Cancer Burden
T-DM1	Trastuzumab emtansine
AJCC	American Joint Committee on Cancer
